# Honey bee hygienic selection impacts virus dynamics of both bees and *Varroa* mites

**DOI:** 10.1016/j.virusres.2026.199715

**Published:** 2026-03-18

**Authors:** Esmaeil Amiri, Somayeh Mehrparvar, Bita Valizadeh, Kaira Wagoner

**Affiliations:** a Department of Agricultural Sciences and Plant Protection, College of Agriculture and Life Sciences, Delta Research and Extension Center (DREC), Mississippi State University, Stoneville, MS, USA; b Department of Biology, University of North Carolina at Greensboro, Greensboro, NC, USA

**Keywords:** Honey bee health, Hygienic behavior, *Varroa* mite, Virus dynamics, Colony mortality

## Abstract

The *Varroa* mite (*Varroa destructor*) has reshaped the viral landscape of honey bee colonies, significantly contributing to colony mortality. In response, *Varroa*-resistant honey bee breeding programs have developed as a promising and sustainable long-term strategy to control *Varroa* mite infestations in managed colonies. These breeding programs drive the coevolution of hygienic bees and *Varroa* mites, however the impact of such coevolution on bee and mite viral dynamics remains poorly understood. To address this gap, we investigated how *Varroa*-resistant traits influence the tripartite interaction among honey bees, *Varroa* mites, and viruses. Two apiaries were established: one in Greensboro, North Carolina, consisting of high and low UBeeO colonies, and another in Stoneville, Mississippi, consisting of Pol-line and Commercial colonies. Worker bees and *Varroa* mites were collected from each colony throughout the beekeeping season and screened for 7 viruses. Hygienic selection significantly reduced the *Varroa* mite infestation level and influenced the dynamics of viruses in worker bees and *Varroa* mites. Specifically, titers of *Varroa*-associated viruses were significantly reduced in worker bees and in mites collected from hygienic colonies. Additionally, hygienic selection altered the co-occurrence patterns and correlations among multiple critically important viruses in mites and worker bees. These findings highlight the value of selective breeding as an effective strategy for improving honey bee health and colony survival and shed light on the complex tripartite relationships between honey bees, *Varroa* mites, and viruses.

## High light

• Varroa-resistant honey bee colonies exhibited reduced titers of multiple Varroa-associated viruses.
• Hygienic selection altered viral prevalence and load in both honey bees and Varroa destructor.
• Virus co-occurrence patterns and correlations differed between hygienic and non-hygienic colonies.
• Results reveal how selective breeding shapes host-vector-virus interactions in honey bee system.

## 1 Introduction

Honey bees (*Apis mellifera* L.) are currently experiencing a major crisis, characterized by widespread colony losses (Nearman et al., 2025), with a projected 34–62% decline in U.S. honey bee colonies in the last decades (Aurell et al., 2024; Giacobino et al., 2024; Steinhauer et al., 2014; vanEngelsdorp et al., 2009). Collective research efforts by the global scientific community have revealed that a complex combination of biotic and abiotic stressors—often varying by region—is implicated in what is now recognized as a multifactorial syndrome (Insolia et al., 2022; Minaud et al., 2024; Neov et al., 2019; Smith et al., 2013; Steinhauer et al., 2018). Among all the identified factors contributing to colony losses, there is a global consensus that the parasitic mite *Varroa destructor* (hereafter *Varroa*) and its associated viruses severely impair honey bee health, weakening honey bee colonies and increasing the likelihood of colony mortality (Dainat et al., 2012; Flores et al., 2021; Francis et al., 2013; Martin, 2001; Morfin et al., 2023; Nazzi and Conte, 2016; Sumpter and Martin, 2004).

Viruses are obligate intracellular parasites that rely entirely on the host’s cellular machinery for replication, and can substantially impact honey bee health and colony survival (McMenamin and Genersch, 2015). *Varroa* are obligate ectoparasites (Rosenkranz et al., 2010; Traynor et al., 2020) that feed upon the fat body and hemolymph of honey bees in both their mobile and reproductive phases. In their mobile phase, *Varroa* move among and feed upon adult bees. In their reproductive phase, *Varroa* enter honey bee brood cells just before cell capping, feed on the developing brood (Han et al., 2024; Ramsey et al., 2019), and lay eggs, some of which develop into mature adults by honey bee eclosion. This indiscriminate feeding behavior of *Varroa* directly compromises individual bee physiology, immunity, and energy reserves (Morfin et al., 2023; Noël et al., 2020), and facilitates the vectoring of several honey bee viruses (Chen et al., 2004; Di Prisco et al., 2011; Posada-Florez et al., 2020; Ryabov et al., 2022; Santillán-Galicia et al., 2014; Shen et al., 2005). Although viruses were present in honey bees before *Varroa* invasion (McMenamin and Genersch, 2015), *Varroa* infestation alters the viral community structure within the honey bee host, transforming covert viral infections into overt, sometimes lethal disease (Doublet et al., 2024; Martin et al., 2012; Mondet et al., 2014; Sumpter and Martin, 2004).

Controlling *Varroa* mites is the primary strategy for managing both *Varroa* infestations and associated virus infections in honey bee colonies (Bubnič et al., 2024; Jack and Ellis, 2021; Locke et al., 2012, 2017), because curative treatments for viral infections are still lacking. While synthetic miticides are commonly used by beekeepers to control *Varroa* populations, the long-time use of these miticides presents several challenges, including the accumulation of chemical residues in hive products (Albero et al., 2023; Premrov Bajuk et al., 2017), and the development of resistance among *Varroa* populations (Kanga et al., 2010; Mitton et al., 2022; Rinkevich, 2020). To overcome these issues, *Varroa*-resistant honey bee breeding programs have been developed as a promising and sustainable solution to control *Varroa* mites and associated viruses in honey bee colonies (Le Conte et al., 2020; O’Shea-Wheller et al., 2022; Rinderer et al., 2010). Several field tests, including the Harbo assay (Harbo, 1996), Freeze-Killed Brood assay (Spivak, 1996), Pin-Killed Brood assay (Newton and Ostasiewski, 1986), and Unhealthy Brood Odor (UBeeO) assay (Wagoner et al., 2021), have been developed to aid in the selection of colonies resistant to mites via hygienic behavior; the detection, uncapping, and removal of unhealthy brood from the colony (Alger et al., 2025; Cremer et al., 2018). Although cannibalization of mite-infested pupae by worker bees may increase the risk of virus infection in individual hygiene-performing bees (Posada-Florez et al., 2021), at the colony level, hygienic behavior suppresses both *Varroa* populations and viral titers (Alger et al., 2025; Erez et al., 2022; O’Shea-Wheller et al., 2022).

Hygienic breeding programs hasten the coevolution of *Varroa* mites and bees, however the impact of such coevolution on viral types and titers within the *Varroa* mite is not well understood. The type, prevalence, and titer of specific viruses may differ between the honey bee and *Varroa* due to differences in viral replication, transmission dynamics, inter-viral competition, or host immune responses (Eliash et al., 2022; Lester et al., 2022; Mondet et al., 2014). *Varroa* mites may also carry their own distinct viruses in addition to harboring bee-associated viruses, further complicating the viral landscape within the mite (Eliash et al., 2022). While many existing studies have focused on *Varroa*-honey bee, or virus-honey bee interactions, the role of *Varroa*-virus interactions and the broader viral ecology within *Varroa* mites in hygienic and non-hygienic colonies remain largely understudied. Therefore, in this study, we investigated the effects of different hygienic selection methods on the tripartite network involving *Varroa* mites, honey bees, and viruses.

## 2 Materials and methods

### 2.1 Experimental design and apiary establishment

Experimental colonies were established in 2022—one in an apiary in Greensboro, North Carolina, and the other in Stoneville, Mississippi.

**The Greensboro apiary** was composed of honey bee packages with hygienic queens (*n* = 22) and nucleus colonies containing *Varroa* Sensitive Hygienic (VSH) daughter queens (*n* = 20) that were purchased from two North Carolina beekeepers (source A and source B) and established in the research apiary of the University of North Carolina at Greensboro, North Carolina in early April and early May 2022, respectively. Neither packages nor nucleus colonies were treated for mites. UBeeO tests were performed to assess the hygienic behavior of each colony in late May, no <7 weeks after release of the caged queens. UBeeO assays were performed as previously described (Alger et al., 2025; Wagoner et al., 2021). Briefly, frames containing capped brood were selected from each colony. A segment of PVC pipe 4 cm in diameter was gently pressed and twisted into an area of capped brood to define the test area (approximately 50 cells), and a photo was taken of the test area at the time of treatment (T_0_). An applicator was used to spray-treat the test area with 0.5 mL of the UBeeO solution. Frames were returned to the colony for 2 h and then recollected. Photos of the test area were taken again at the end of the testing period (T_2_). Photos were used to determine the number of capped cells that fell ≥50% inside the test area at T_0_ and T_2_ for each colony. UBeeO scores were calculated using the equation: UBeeO Score = 1 - (capped cells at T_2_/ capped cells at T_0_) * 100. Colonies that scored ≥ 60% were classified as hygienic or “high UBeeO” and colonies that scored <60% were classified as non-hygienic or ”low UBeeO” (Wagoner et al., 2021).

The 7 highest and 7 lowest UBeeO scoring colonies from each source were initially included in the study (*n* = 28 total). One colony from source A and two colonies from source B were found queen-less and/or swarmed early in the study, and thus were excluded from analysis, leaving 7 high-scoring colonies and 6 low-scoring colonies from source A, and 5 high-scoring colonies and 7 low-scoring colonies from source B. An additional high-scoring colony was added to the study to balance sample sizes (13 high and 13 low UBeeO scoring colonies in total). This colony was selected from the university apiary, as no other high-scoring colonies were available from source B.

**The Stoneville apiary** was established by 20 honey bee packages of Russian honey bees (Strange et al., 2008), each containing ∼3 lb of young worker bees in May 2022. The source colonies of these package honey bees were treated one month before making packages to minimize *Varroa* mite infestation. Ten of these packages were headed by young queens derived from the same Russian honey bee stock (commercial stock), while the remaining ten were headed by young Pol-line queens (a selectively bred line (Pollinator line) for *Varroa* Sensitive Hygienic (VSH) behavior) obtained from the Honey Bee Breeding, Genetics, and Physiology Laboratory (USDA-ARS, Baton Rouge, LA). Before package installation, a random sample of 50 worker bees was collected from each package to assess viral infection diversity and titer. At the time of package installation, colonies were randomly located 2 m from each other to prevent worker bee drifting throughout the experiment. Though the surrounding area provided ample amounts of nectar and pollen from wild flowers and agricultural crops, each colony received 2 liters of sugar syrup (50% sucrose solution, w/v) every third day for four weeks to ensure experimental acclimation and encourage comb construction. One queen from each source was superseded soon after installation, so those two colonies were excluded from the experiment, and sampling was performed from the 18 remaining colonies.

### 2.2 Sample collection

Honey bee and *Varroa* mite samples were collected from each colony in each apiary to evaluate the titer and type of viruses. At the Greensboro apiary, sampling was conducted over four months at the end of the first week of June, July, August, and September. At the Stoneville apiary, initial sampling in May and June was conducted to establish baseline viral titers during the period of genetic transition following queen replacement. Subsequent samplings were carried out during the third week of July, August, September, and October to monitor *Varroa* mite infestation levels and viral infection dynamics.

Bee samples were collected by dragging a 50 mL Falcon Tube (∼100 worker honey bees) down the center of a frame containing open brood cells. Mite counts (adult infestation levels) were assessed by collecting an additional sample of bees from a separate frame containing open brood cells. Approximately 300 bees were brushed into a glass jar and capped with a mesh lid. Mite samples for viral analysis were collected using the powder sugar shake method, as described previously (Macedo et al., 2002). Briefly, several tablespoons of powdered sugar were added to the jar through the mesh to induce honey bee grooming behavior. The jar was agitated lightly to cover all the bees in the sugar and then set aside for approximately 1 min. The jar was then inverted over a white tub and shaken so that the groomed mites fell into the tub and could be collected in 2 mL micro-centrifuge tubes. Once no more mites were falling from the jar, all the mites were counted. To improve the accuracy of mite infestation counts, bee samples were then processed via a soapy water rinse. Soapy water was added to each jar, bees were poured into it, and then rinsed in a double sieve with mesh apertures of ∼ 3 mm and ∼ 0.5 mm to isolate bees and mites, respectively. For each colony, the number of mites (sugar shake + soapy water rinse) and bees were counted, and the percent mite infestation was calculated using the equation: Mite infestation = (total # mites / total # of bees) * 100. All bee and mite samples collected for viral analysis were frozen within 2 h and stored at −80 °C until analysis.

### 2.3 Sample handling and preparation

All samples collected in Greensboro were sent to the Center for Pollinator Health at the Delta Research and Extension Center in Stoneville, MS, to be analyzed alongside those collected from the Stoneville apiary. For each monthly sampling, worker bees collected from each colony were inspected for *Varroa* mites on a laboratory cold plate (TecaLab Products, Chicago, IL, USA). Any mites observed were carefully removed, and a pooled sample of 50 worker bees per colony was prepared for RNA extraction. Mites collected monthly from each colony (*n* = 10 mites/colony if available) were individually placed in a labeled microcentrifuge tube, indicating the collection date and colony of origin, and stored in the −80 °C freezer until RNA extraction. Honey bee worker samples were subsequently freeze-dried using a Triad™ freeze dryer (Labconco Corporation, Kansas City, MO, USA) for two weeks at pressure 0.05 hPa and temperature −50 °C. After lyophilization, four metal beads (2.4 mm, PerkinElmer Co., USA) were added to each sample tube and samples were homogenized using a Bead Ruptor Elite (OMNI International Inc.). Approximately 10–15 mg of the powdered material was used to extract RNA.

### 2.4 RNA extraction, cDNA conversion, and real-time-qPCR

Total RNA was extracted from each sample (pooled worker honey bees and individual *Varroa* mites) using the MagMAX mirVanna Total RNA Isolation Kit (Thermo Fisher Scientific, Baltics, UAB) in a KingFisher magnetic extractor (KingFisher Duo Prime, Thermo Fisher Scientific) following the manufacturer’s protocol with minor modifications. RNA concentration and purity were measured using a NanoDrop One Microvolume UV–Vis Spectrophotometer (Thermo Fisher Scientific, MA, USA). The RNA concentration from single *Varroa* mites were generally low; therefore, RNA samples were adjusted to 25 ng/μL, while RNA from worker bee samples was adjusted to 50 ng/μL using the elution buffer provided in the extraction kit. Complementary DNA (cDNA) was synthesized using the High-Capacity cDNA Reverse Transcription Kit (Applied Biosystems, Foster City, CA, USA). Each reaction included 500 ng RNA template (10 μL) and 10 μL of the supplied cDNA master mix, and was incubated in a thermocycler (Mastercycler® nexus Series Thermal Cyclers, Eppendorf, Hamburg, Germany) under the following conditions: 10 min at 25 °C, 120 min at 37 °C, and 5 min at 85 °C. The resulting cDNA was diluted 10-fold in molecular-grade water for subsequent qPCR analysis.

The qPCR analysis was performed in duplicate on 384-well plates using a QuantStudio 6 Pro system (Applied Biosystems, USA). Reactions were carried out using PowerUp SYBR Green Master Mix (Thermo Fisher Scientific Baltics UAB, Vilnius, Lithuania) in a total volume of 12 μL with final primer concentrations of 0.4 μM. Previously validated primers were used to detect seven viruses including six honey bee viruses (deformed wing virus type A and B (DWV-A, DWV-B), sacbrood virus (SBV), black queen cell virus (BQCV), Lake Sinai viruses (LSVs), and bee rhabdovirus-1 (BRV-1) formerly known as Apis rhabdovirus-1) and a *Varroa* mite virus (VDV2) (Supplementary Table S1). Reference genes RPS5 (for honey bees) and β-actin (for *Varroa* mites) were included as internal controls to confirm the integrity of sample processing and the functionality of the assay. Each qPCR plate included a ten-fold serial dilution of synthesized viral amplicons as a positive control, RNase-free water as a No Target Control (NTC), and a No Reverse Transcriptase (NRT) control to rule out genomic DNA contamination (Bustin et al., 2009). The thermal cycling conditions were initialized for 10 min at 95 °C, followed by 40 cycles of 95 °C for 15 s and 60 °C for 1 min. A final melt curve dissociation analysis was performed to confirm the specificity of amplification products. Samples were considered positive for a target if the melt curve matched that of the corresponding positive controls and the Cq value was ≤35. Based on the standard curves, a Cq value of 35 was chosen as a cut-off because the standard curves were no longer linear after cycle 35. Viral copy numbers were determined by absolute quantification, based on standard curves generated from serial dilutions of known concentrations of viral amplicons, as described before (Amiri et al., 2015).

### 2.5 Statistical analysis

#### 2.5.1 Statistical packages

All statistical analyses were performed using R. Data handling, organization and visualization was performed using the packages brms (Bürkner, 2017), bayestestR (Makowski et al., 2019), bayesplot (Gabry et al., 2019), readxl (Wickham and Bryan, 2019), emmeans (Lenth, 2018), ggplot2 (Wickham, 2016), dplyr (Wickham et al., 2025), tidyverse (Wickham, 2017), patchwork (Pedersen, 2025a), cowplot (Wilke, 2025), ggpubr (Kassambara, 2025), stringr (Wickham, 2023), cooccur (Griffith et al., 2016), corrr (Ruiz et al., 2019), corrplot (Wei and Simko, 2024), tidygraph (Pedersen, 2025b), reshape2 (Wickham, 2007), ggfortify (Tang et al., 2016), broom (Robinson et al., 2025), and agricolae (Mendiburu, 2019).

#### 2.5.2 Statistical methods

To assess colony *Varroa* infestation and viral loads in honey bee workers and *Varroa* mites, we applied a Bayesian generalized linear mixed model using a hurdle lognormal distribution. This modeling approach was selected due to the right-skewed *Varroa* mite distribution, the high frequency of zeros (i.e., samples in which no virus was detected), and the right-skewed distribution of viral load data. In the model, the interaction between colony hygienic behavior and sampling date was considered as a fixed effect, while colony identity was treated as a random effect. In addition to the virus-specific analysis, total viral load (the sum of all quantified viruses per colony) was analyzed separately for worker bees and *Varroa* mites in each experimental apiary to evaluate the effect of hygienic behavior on the total virus titer in each colony and *Varroa* mite. For this analysis, we used a lognormal distribution instead of a hurdle model, while retaining the same structure of fixed and random effects. Estimated marginal means and contrasts between groups within each date were calculated separately for worker honey bee samples and *Varroa* mites. The model outputs were computed at 95% credible interval, and posterior distributions were examined to assess statistical evidence.

Finally, a probabilistic co-occurrence model (Veech, 2013) was used to evaluate the likelihood of virus co-occurrence in honey bees and *Varroa* mites in experimental colony groups in each apiary. Using binary prevalence data for each virus in worker bees and *Varroa* mites, the model assessed how each pair of viruses co-occurred within the same samples either more or less frequently than expected under a random distribution. To visualize these patterns, co-occurrence networks were constructed in which nodes represent individual viruses, node size represents virus prevalence, edges indicate significant associations, edge color denotes co-occurrence direction, and widths represent the strength of the correlations.

## 3 Results

### 3.1 *Varroa* mite infestation level

**Fig. 1:**
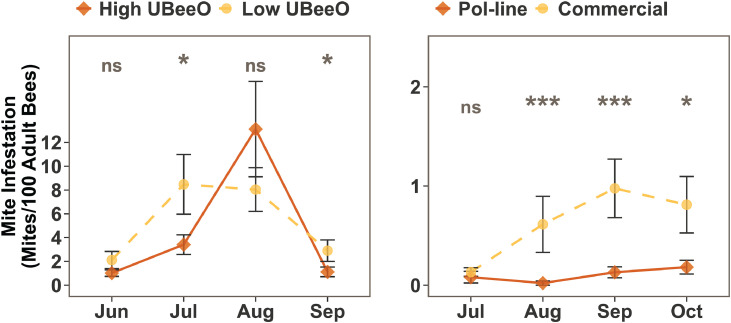
**Seasonal dynamics of *Varroa* mite infestation in honey bee colonies at two apiaries.** The left graph shows mean mite infestation levels (mite per 100 bees ± SE) from June to September in high and low UBeeO colonies at the Greensboro apiary. The right graph shows infestation levels from July to October in Pol-line and Commercial colonies at the Stoneville apiary. Astrix indicate significant differences between groups at each time point based on Bayesian posterior distributions as: *p* < 0.05 (*), *p* < 0.01 (**), *p* < 0.001 (***), and ns= not significant. In both apiaries, colonies from each group initially exhibited similar level of *Varroa* infestation. However, as the experiment progressed, *Varroa* mite levels tended to be lower in the high UBeeO and Pol-line colonies compared to the low UBeeO and Commercial colonies.

*Varroa* mite infestation levels were higher throughout the experiment at the Greensboro apiary compared to the Stoneville apiary (Fig. 1). Early in the season, *Varroa* mite levels in Greensboro did not differ significantly between high and low UBeeO colonies (median ratio: 0.697, 95% CI: 0.262–1.830, *p* = 0.496). However, mite levels in high UBeeO colonies were significantly lower than mite levels of low UBeeO colonies by July (median ratio: 0.410, 95% CI: 0.170–0.952, *p* = 0.042). The average *Varroa* mite infestation in the (surviving) low UBeeO colonies did not differ from that of high UBeeO colonies in August (median ratio: 0.932, 95% CI: 0.359–2.423, *p* = 0.841). However, by September, *Varroa* mite infestation levels in high UBeeO colonies were again significantly lower than those in low UBeeO colonies (median ratio: 0.367, 95% CI: 0.144–0.961, *p* = 0.042).

A similar trend was observed between Pol-line and commercial colonies in Stoneville, but average mite infestation never exceeded 2%. Although no significant differences in *Varroa* infestation levels were observed in July (median ratio: 0.583, 95% CI: 0.098–3.083, *p* = 0.357), the infestation levels differed significantly between the two groups in the following months (median ratio: 0.057, 0.170, 0.256, 95% CI: 0.005–0.519, 0.040–0.563, 0.069–0.798, *p* = 0.001, 0.001, and 0.014 in August, September, and October respectively).

### 3.2 Viral types and titer

#### 3.2.1 The Greensboro apiary

**Fig. 2:**
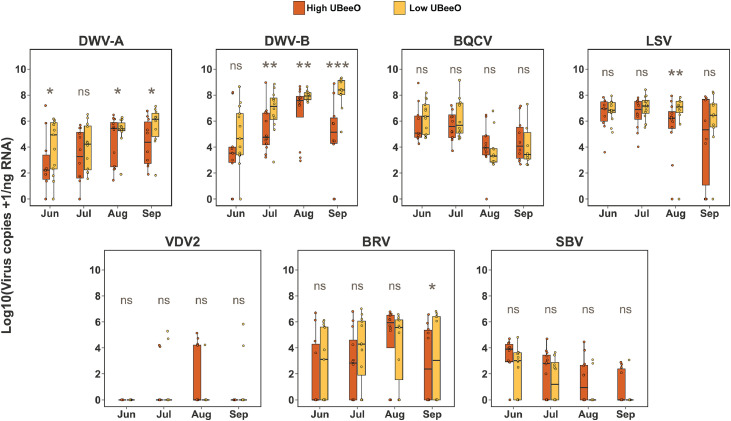
**Temporal pattern of viral titers in worker honey bees from high and low UBeeO colonies at the Greensboro apiary**. Boxplots show Log10 transformed viral titer for seven viruses (DWV-A, DWV-B, BQCV, LSVs, VDV2, BRV-1, and SBV). Samples were collected monthly from June to September from colonies headed by high UBeeO queens (Orange boxes) and low UBeeO queens (Yellow boxes). Statistically significant differences were assessed using Bayesian posterior distributions *p* < 0.05 (*), *p* < 0.01 (**), *p* < 0.001 (***), and ns= not significant as estimated via Bayesian pairwise comparisons. High UBeeO colonies exhibited consistently lower titers of DWV-B across three months (July-September), and lower levels of DWV-A, LSVs, BRV-1 and SBV in some months suggesting an association between colony genotype and reduced virus level.

Viral titer data from worker bees collected at the Greensboro apiary showed a distinct viral titer pattern (Fig. 2). Overall, titers of DWV-A and DWV-B increased from June to September in both high and low UBeeO colonies. However, high UBeeO colonies consistently exhibited lower DWV titers compared to low UBeeO colonies. Specifically, DWV-A titers were significantly lower in high UBeeO colonies in June (median ratio: 0.086, 95% CI: 0.003–2.888, *p* = 0.021), August (median ratio: 0.146, 95% CI: 0.005–4.064, *p* = 0.04), and September (median ratio: 0.053, 95% CI: 0.002–1.925, *p* = 0.36), though no difference was observed in July (median ratio: 0.586, 95% CI: 0.019–19.048, *p* = 0.642). DWV-B titers were also lower in high UBeeO colonies across the sampling period, with statistically significant differences observed in July (median ratio: 0.037, 95% CI: 0.002–0.955, *p* = 0.006), August (median ratio: 0.053, 95% CI: 0.002–1.365, *p* = 0.005), and September (median ratio: 0.007, 95% CI: 0.000–0.249, *p* = 0.001). Although no statistically significant differences were observed for BQCV and SBV throughout the entire experiment, both viruses showed a downward trend in both groups in August and September. A significant difference between viral titers of high and low UBeeO colonies was observed for LSVs in August (median ratio= 0.105, 95% CI: 0.012–0.987, *p* = 0.009), and for BRV-1 in September (median ratio= 0.122, 95% CI: 0.003–4.654, *p* = 0.043). VDV2 was detected periodically with no significant differences observed between low and high UBeeO colonies.

**Fig. 3:**
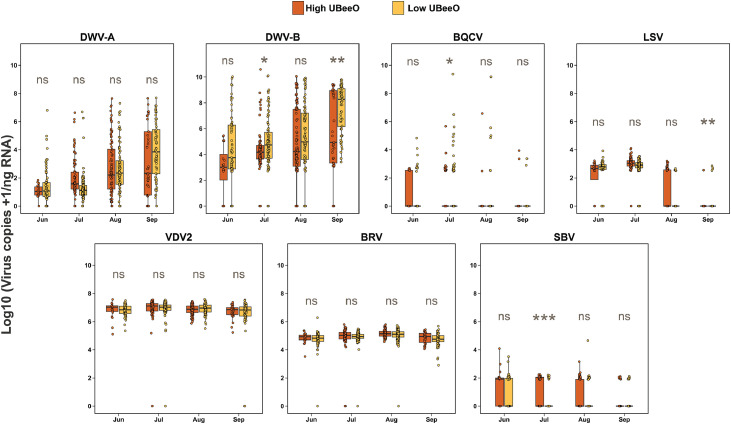
**Temporal pattern of viral titers in *Varroa* mites from high and low UBeeO colonies at the Greensboro apiary**. Sampling took place from July to September, and samples were tested for 7 viruses including: DWV-A, DWV-B, BQCV, LSVs, SBV, BRV-1, and VDV-2. Statistically significant differences were assessed using Bayesian posterior distributions *p* < 0.05 (*), *p* < 0.01 (**), *p* < 0.001 (***), and ns= not significant as estimated via Bayesian pairwise comparisons.

Viral titers of *Varroa* mites collected at the Greensboro apiary were also analyzed (Fig. 3) for comparison to those of worker bees. Titers of both DWV-A and DWV-B increased in *Varroa* mites from June to September. However, no statistically significant differences were observed in DWV-A loads between *Varroa* mites collected from high and low UBeeO colonies throughout the experiment. In contrast, DWV-B titers in *Varroa* mites from high UBeeO colonies tended to be lower than those from low UBeeO colonies, with statistically significant differences observed in July (median ratio: 0.196, 95% CI: 0.023–1.675, *p* = 0.030) and September (median ratio: 0.085, 95% CI: 0.028–2.004, *p* = 0.007), but not in June or August. BRV-1 and VDV2 titers were similar between mites collected from high and low UBeeO colonies, with no statistically significant differences. As the experiment progressed BQCV, SBV and LSVs become less prevalent, showing a general decrease in viral titer and significant differences were detected only in July for BQCV (median ratio: 0.191, 95% CI: 0.020–1.915, *p* = 0.037) and SBV (median ratio: 6.499, 95% CI: 2.432–18.90, *p* = 0.001), and in September for LSVs (median ratio: 0.047, 95% CI: 0.002–1.058, *p* = 0.003).

#### 3.2.2 Stoneville apiary

**Fig. 4:**
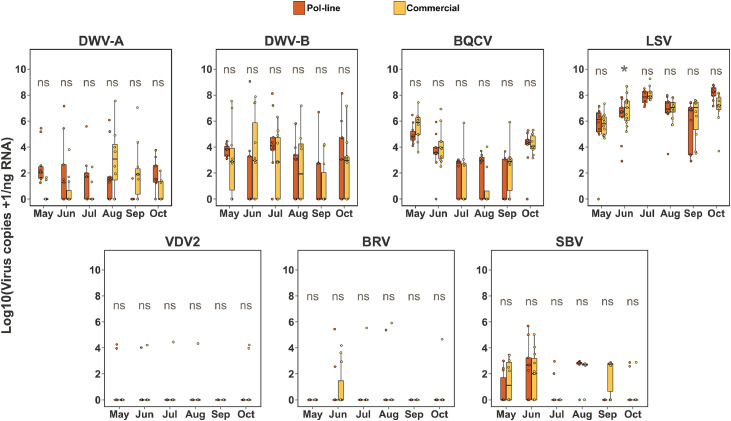
**Temporal pattern of viral titers in worker honey bees collected from Pol-line and commercial colonies in the Stoneville apiary**. Boxplots show Log10 transformed viral titer for seven honey bee viruses (DWV-A, DWV-B, BQCV, LSVs, VDV2, BRV-1, and SBV) from May to October. Orange boxes indicate samples from Pol-line colonies, while yellow boxes represent the Commercial colonies. Each panel shows viral titers over time, and statistical comparison between Pol-line and Commercial colonies. Statistically significant differences were assessed using Bayesian posterior distributions *p* < 0.05 (*), *p* < 0.01 (**), *p* < 0.001 (***), and ns= not significant as estimated via Bayesian pairwise comparisons.

Unlike the worker bee samples collected at the Greensboro apiary, DWV-A and DWV-B titers did not show an increasing trend over time in the Stoneville apiary (Fig. 4), and no significant differences were observed between Pol-line and commercial colonies throughout the experiment. The titer of LSVs was higher than that of other viruses, with a statistically significant difference observed only in June (median ratio: 0.216, 95% CI: 0.021–2.314, *p* = 0.049). BQCV and SBV titers generally declined toward the end of the experiment with no significant differences between Pol-line and commercial colonies. While BQCV titers increased in October compared to the three previous months, no viral differences were detected between Pol-line and commercial colonies. BRV-1 and VDV2 were detected periodically throughout the experiment, with no consistent trends observed for either virus.

**Fig. 5:**
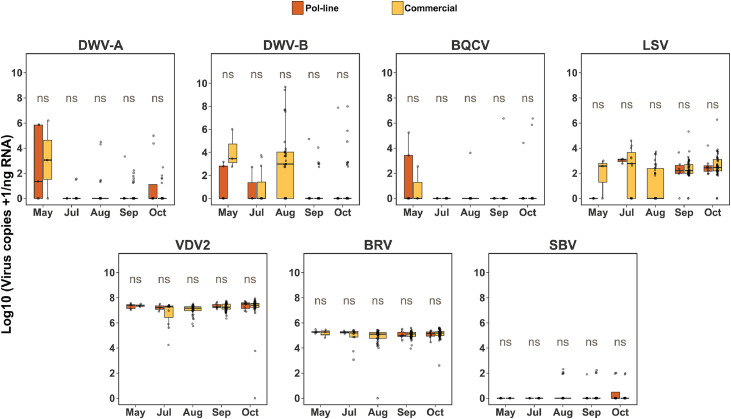
**Temporal patterns of viral titer in *Varroa* mite samples collected from Pol-line and commercial colonies in the Stoneville apiary.** Boxplots show monthly viral titers of 7 viruses including: DWV-A, DWV-B, BQCV, LSVs, SBV, BRV-1, and VDV-2 from May to October. Each panel compares virus titers between *Varroa* mites collected from Pol-line colonies (Orange) and Commercial colonies (Yellow color). Statistically significant differences were assessed using Bayesian posterior distributions *p* < 0.05 (*), *p* < 0.01 (**), *p* < 0.001 (***), and ns= not significant as estimated via Bayesian pairwise comparisons. DWV-A, DWV-B, and BQCV showed higher titers early in the season with apparent declines by late summer, while VDV2, BRV-1, LSVs and SBV remained largely stable throughout the sampling variable.

No significant differences in viral titer were observed between *Varroa* mite samples collected from Pol-line and commercial colonies in the Stoneville apiary (Fig. 5). Virus titers for DWV-A, DWV-B, SBV, and BQCV declined toward the end of the experiment, while titers of LSVs, BRV-1, and VDV2 remained relatively unchanged.

#### 3.2.3 Total viral titer in worker honey bees and *Varroa* mites

**Fig. 6:**
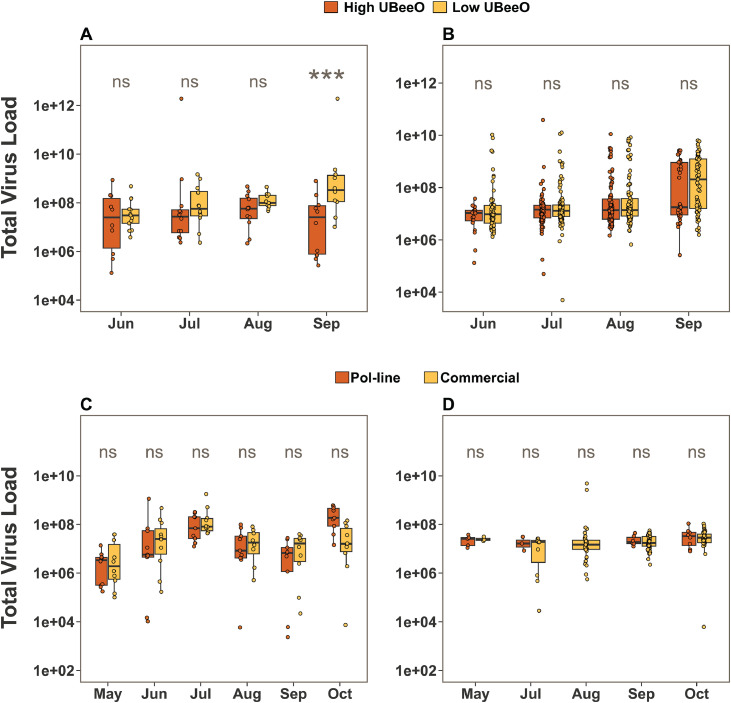
**Effect of genetic stock on total viral titer (the sum of all 7 quantified viruses) in the investigated samples from both apiaries over the course of experiment**; A) Worker honey bee sampled from high and low UBeeO colonies in Greensboro apiary, B) *Varroa* mite samples from high and low UBeeO colonies in Greensboro apiary, C) Worker honey bees from Pol-line and Commercial colonies in Stoneville apiary, and D) *Varroa* mite samples from Pol-line and Commercial colonies in Stoneville apiary. Statistically significant differences were assessed using Bayesian posterior distributions *p* < 0.05 (*), *p* < 0.01 (**), *p* < 0.001 (***), and ns= not significant as estimated via Bayesian pairwise comparisons. Significant differences were observed in September between high and low UBeeO colonies (Panel A).

Total viral titer in worker honey bees collected from the Greensboro apiary tended to be higher in low UBeeO colonies compared to high UBeeO colonies (Fig. 6-A), with a statistically significant difference observed in September (median ratio: 0.022, 95% CI: 0.002–0.202, *p* = 0.001). In contrast, no significant differences were observed in the total viral titer between *Varroa* mites collected from high and low UBeeO colonies (Fig. 6-B). Overall viral titer in worker honey bees from Pol-line and Commercial colonies at the Stoneville apiary did not differ significantly (Fig. 6-C), and the same trend was observed in the corresponding *Varroa* mite samples (Fig. 6-D).

### 3.3 Co-occurrence and correlation of viruses

**Fig. 7:**
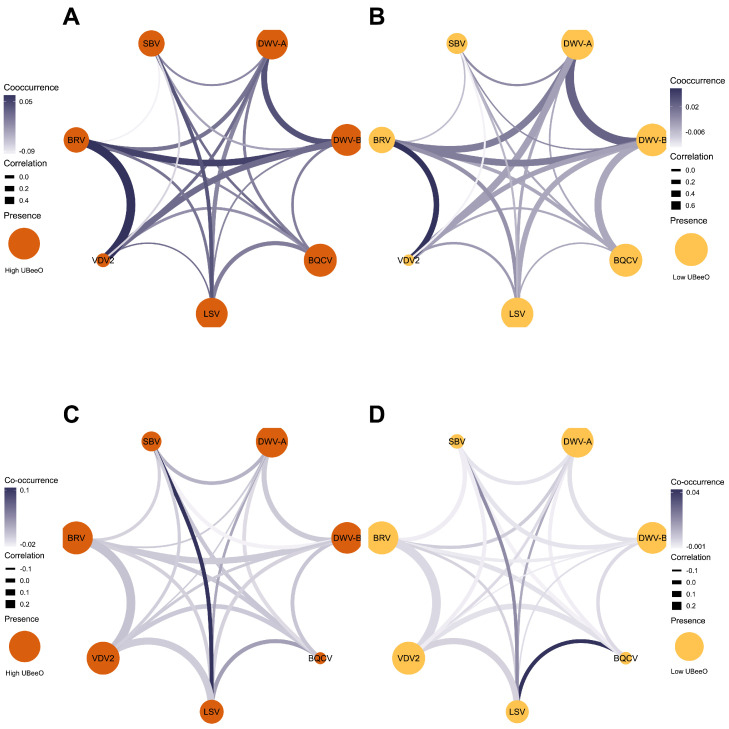
**Co-occurrence and correlation networks of honey bee viruses in colonies with different UBeeO levels in the Greensboro apiary.** (A) worker honey bees in high UBeeO colonies, (B) worker honey bees in low UBeeO colonies, (C) *Varroa* mites in high UBeeO colonies, and (D) *Varroa* mites in low UBeeO colonies. Each node represents a virus, with node color indicating high UBeeO (Orange), or low UBeeO (Yellow). The size of nodes represents the prevalence of viruses. The thickness of the edges represents the strength of correlation (based on virus abundance), while the color intensity of edges indicates co-occurrence values.

**Fig. 8:**
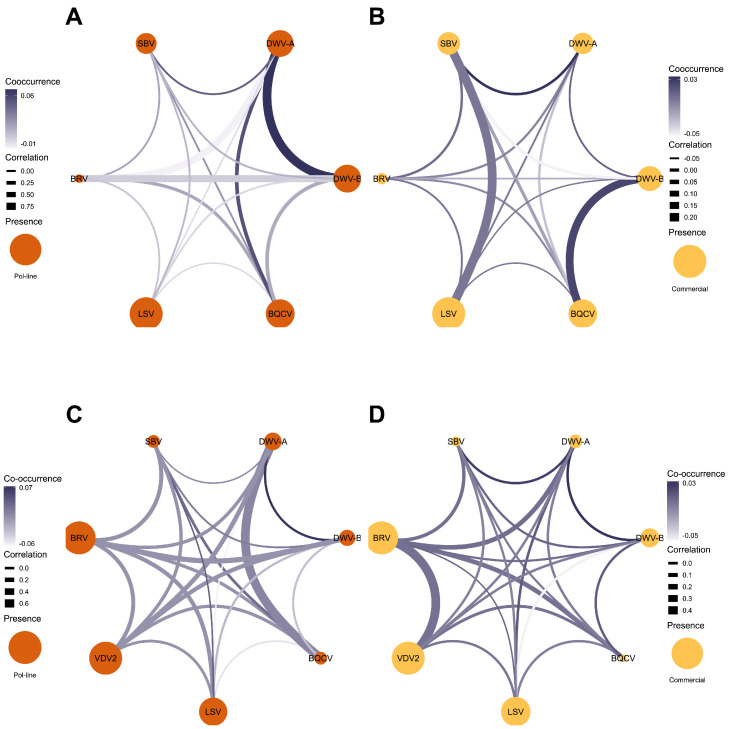
**Co-occurrence and correlation network of viruses in worker honey bees and *Varroa* mites collected from Pol-line and Commercial colonies in the Stoneville apiary.** (A) worker honey bees in Pol-line colonies, (B) worker honey bees in Commercial colonies, (C) *Varroa* mites in Pol-line colonies, and (D) *Varroa* mites in Commercial colonies. The size of orange nodes represents the prevalence of viruses. Co-occurrence represented as deviation from random expectations is presented as edge color, with an increase in dark blue color representing an increase in co-occurrence, while correlation of viral load (in copy number) is presented as the edge thickness, with thicker lines symbolizing a higher degree of correlation.

The prevalence of viruses in the investigated samples (supplementary table S2 and S3) is indicated by the size of nodes in Figs. 7& 8. In Greensboro, the virus prevalence in worker bees from high UBeeO colonies ranged from 15.9% (VDV2) to 97.7% (BQCV) (Fig. 7-A). In low UBeeO colonies, virus prevalence ranged from 10.9% (VDV2) to 100% (BQCV) (Fig. 7-B). In *Varroa* mite samples, the virus prevalence showed a reverse trend with lower BQCV and higher VDV2. Virus prevalence in mites ranged from 11.8% (BQCV) to 99.6% (VDV2) in high UBeeO colonies (Fig. 7-C) and 13.1% (BQCV) to 99.1% (BRV-1 and VDV2) in low UBeeO colonies (Fig. 7-D). In high UBeeO colonies, several viruses, including BRV-1/VDV2, BRV-1/DWV-B, DWV-A/DWV-B, and LSVs/SBV, showed a higher co-occurrence in worker bees (Fig. 7-A), whereas in low UBeeO colonies, only DWV-A/DWV-B and BRV-1/VDV2 indicated a cluster of co-occurrences (Fig. 7-B). The co-occurrence in *Varroa* mite samples showed a cluster between LSVs/SBV, LSVs/DWV-A, and LSVs/BQCV in high UBeeO colonies (Fig. 7-C), whereas in low UBeeO colonies, co-occurrence was stronger between LSVs/SBV and LSVs/BQCV than other viruses (Fig. 7-D). Significant positive correlation was observed between BRV-1 with VDV2 and DWV-B (*r* = 0.001 and 0.04, respectively) in worker bee samples from high UBeeO colonies (Fig. 7-A), and between DWV-A/DWV-B, DWV-B/BQCV, and BRV-1/VDV2 (*r* = 0.001, 0.001, and 0.03, respectively) in worker bees from low UBeeO colonies (Fig. 7-B). Positive and significant correlations were observed between VDV-2 with LSVs and BRV-1 (*r* = 0.023 and 0.001, respectively) in *Varroa* mite samples from high UBeeO colonies (Fig. 7-C). Also, positive and significant correlations were observed between DWV-B/LSV, BWV-B/VDV-2, and VDV2/BRV-1 (*r* = 0.032, 0.023, and 0.001, respectively, Fig. 7-D).

In Stoneville apiary, the prevalence of viruses ranged from 5.5% (BRV-1) to 98.2% (LSVs) in Pol-line colonies (Fig. 8-A), whereas in the commercial colonies they ranged from 10.2% (BRV-1) to 100% (LSVs, Fig. 8-B). Due to very few positive samples for VDV2, we did not add these numbers in the prevalence calculation. The prevalence of viruses in *Varroa* mite samples ranged from 13.6% (BQCV and SBV) to 100% (VDV2 and BRV-1) in Pol-line colonies (Fig. 8-C) and 3.7% (BQCV) to 99.3% (VDV2 and BRV-1) in commercial colonies (Fig. 8-D). Viral co-occurrence was more likely between DWV-A/DWV-B, DWV-A/BQCV, DWV-A/SBV, and BQCV/SBV in honey bee samples in the Pol-line colonies (Fig. 8-A), whereas in Commercial colonies DWV-A/SBV, and DWV-B/BQCV were more likely to co-occur (Fig. 8-B). In *Varroa* mite samples collected from Pol-line colonies, co-occurrence of DWV-A/DWV-B, LSVs/SBV, and BQCV/SBV was more likely (Fig. 8-C), while in Commercial colonies DWV-A/DWV-B, and DWV-A/SBV were more likely to co-occur (Fig. 8-D). While correlations among viruses in worker bee samples varied, significant positive correlations were observed between DWV-A/DWV-B, DWV-A/BRV-1, and DWV-B/BRV-1 (*r* = 0.001 for all) in pol-line colonies (Fig. 8-A), whereas no significant correlation was observed among viruses in worker bees from Commercial colonies (Fig. 8-B). In *Varroa* mite samples collected from Pol-line colonies, only the correlation between DWV-A/BQCV was significant (*r* = 0.002, Fig. 8-C), while in the *Varroa* samples from commercial colonies, only the correlation between VDV-2/BRV-1 was significant (*r* = 0.001, Fig. 8-D).

## 4 Discussion

The *Varroa* mite and its associated viruses are the primary causes of honey bee colony mortality (Francis et al., 2013; Martin, 2001; McMenamin and Genersch, 2015; Nazzi et al., 2012; Traynor et al., 2020). Several integrated pest management strategies have been proposed to keep *Varroa* mite levels below economic threshold (ET) and reduce honey bee colony virus loads (Jack and Ellis, 2021; Locke et al., 2012, 2017; Rosenkranz et al., 2010). One promising solution to effectively control *Varroa* mites and related viruses is the breeding of *Varroa*- and disease-resistant honey bees (Büchler et al., 2010; Erez et al., 2022; Rinderer et al., 2010; Spivak, 1996). Our study provides compelling evidence that hygienic selection of honey bee colonies significantly influences both *Varroa* mite infestation and colony viral dynamics, particularly with respect to DWV-A and DWV-B. The consistent differences observed between selected (high UBeeO and Pol-line) and unselected for hygiene (low UBeeO and Commercial) colonies across two geographically distinct apiaries confirm previous research findings related to hygienic selection for pest and disease resistance and highlight the value of selective breeding as a strategy for improving honey bee health and colony survival (O’Shea-Wheller et al., 2022; Toufailia et al., 2014).

### 4.1 Influence of honey bee stock on *Varroa* mite infestation

Overall, mite infestation levels were significantly lower in high UBeeO and Pol-line colonies than in low UBeeO and Commercial colonies, demonstrating the beneficial effects of hygienic selection on colony *Varroa* mite populations. Mite levels also differed between apiaries, with markedly fewer mites at the Stoneville location. This apiary difference was likely due to the use of Russian honey bees, which are historically known to control *Varroa* mite populations, or to treatment applied to source colonies prior to package preparation, before the experiment; however, climatic variation may also have contributed (Harris et al., 2003; Korená Hillayová et al., 2022; McAfee et al., 2024), or differences in mite pressure from nearby colonies between the sites (DeGrandi-Hoffman et al., 2016). While the colonies in the Greensboro apiary were established from nucleus colonies or package bees installed in boxes with drawn combs, the package bees in the Stoneville apiary were installed in boxes with foundation frames, which required additional time to build combs and initiate brood rearing (Mortensen et al., 2025). This delay may have postponed the growth of the *Varroa* mite population in the Stoneville apiary. In both locations, the mite population increased as the season progressed, reaching its peak in August in Greensboro and September in Stoneville. This offset of mite population peaks between apiaries is consistent with expectations, given the extended brood production associated with the warmer climate in Stoneville. Though infestation levels never exceeded 2% in Stoneville, statistically significant differences were detected between Pol-line and Commercial colonies, with fewer mites present in colonies selected for *Varroa* resistance. Differences in mite infestations between groups of colonies in each location demonstrate the impact that queen genetics can have on colony resistance to pests and diseases over time. Three low UBeeO colonies with June infestation rates above 2% were lost at the Greensboro apiary in July, likely due to high *Varroa* loads. The collapsing colonies were robbed out by other colonies in the apiary, which is a likely explanation for the temporary increase in mite counts for high UBeeO colonies in August (DeGrandi-Hoffman et al., 2016). Mite loads in high UBeeO colonies returned to lower levels by September, likely due to hygienic removal of mites once they entered the brood and were subjected to hygienic behavior.

### 4.2 Viral load dynamics and vector-host relationship

The viral profile of bees in the Greensboro apiary mirrored the differences observed in mite infestation, with high UBeeO colonies consistently exhibiting lower viral titers than low UBeeO colonies, particularly for DWV-A and DWV-B. This finding is consistent with the knowledge that DWV-A and -B are vectored by and replicated within *Varroa* mites (Damayo et al., 2023; Gisder et al., 2009; Norton et al., 2021; Posada-Florez et al., 2020; Ryabov et al., 2022). Virus loads of mites further mirror this relationship, indicating that the impacts of hygiene on virus loads extends beyond honey bees to mites. Interestingly, the lack of difference in mite infestations for high and low UBeeO colonies in August was not mirrored in August *bee* virus titers but was mirrored in August *mite* DWV-B titers. This supports the theory that mites from collapsing low UBeeO colonies caused temporary spikes in the populations of mites with high DWV levels in high UBeeO colonies and suggests that such temporary spikes may not have major implications for honey bee colony viral loads. Furthermore, the rapid recovery of high UBeeO colonies suggests that bees selected for hygienic behavior may be capable of suppressing substantial mite drift from collapsing colonies. This spike/recovery dynamic has implications for *Varroa*-resistance selection programs based solely on measuring adult bee infestation, indicating that follow-up assessments may be useful in distinguishing temporary mite population spikes caused by drift from more sustained colony susceptibility to mites. In general, the mechanisms driving differences in mite viral loads between high and low UBeeO colonies are not yet understood. It is likely that lower virus loads in bees from high UBeeO colonies lead to decreased spread of viruses from bees to mites during mite parasitization. However, since higher virus loads in mites are likely associated with increased honey bee brood stress, it is also possible that brood infested by mites with high viral loads send out stronger stress signals, making them more likely to be targeted by worker bees for hygienic uncapping and removal. This signal-strength hypothesis would explain why the highly virulent DWV-B was found to be significantly lower in mites from high UBeeO colonies. Another possibility is that virus loads increase in mites over their lifetime (Ryabov et al., 2022), and that, given their greater likelihood of detection, mites in high UBeeO colonies have a shorter lifespan and thus tend to have lower viral loads. This lifespan hypothesis would explain our findings with DWV-B, BQCV, and LSV, and the similar (though non-significant) trend seen for DWV-A. Further research is required to test these hypotheses, which are not mutually exclusive, and to better understand the complex relationships between honey bees, mites, and viruses.

Our results confirm previous findings that reduced mite loads in high UBeeO colonies limit virus titers (Alger et al., 2025). *Varroa* mites are well known for their role in horizontal virus transmission; therefore, low *Varroa* mite populations in high UBeeO colonies may limit the primary route of horizontal virus transmission between bees (Bubnič et al., 2024; Locke et al., 2012). Interestingly, while DWV-B titers of mites from high UBeeO colonies were significantly lower than those from low UBeeO colonies, DWV-A loads of mites did not differ significantly between high and low UBeeO colonies. This finding is consistent with DWV-B’s known replication in the mite vector and confirms previous evidence that DWV-B is more tightly coupled to *Varroa* transmission dynamics than DWV-A, which may be more reliant on host-mediated proliferation (Gisder et al., 2009; Ryabov et al., 2022). These findings also highlight the importance of simultaneously analyzing viral titers in both hosts and vectors to understand transmission ecology (Eliash et al., 2022; Lester et al., 2022).

Viral titers in worker bees and mites at the Stoneville apiary did not vary significantly between Pol-line and Commercial colonies. This is likely a result of the uniformly low *Varroa* mite infestation, which did not exceed the economic treatment threshold of 3% for either Pol-line or Commercial colonies. While results from both sites indicate the benefits of selecting for honey bee mite and disease resistance, stronger statistical support for differences measured in the Greensboro apiary compared to the Stoneville apiary also highlights the likelihood of within-stock variation in colony hygienic behavior. Such within-stock variation is expected, especially for open-mated queens. Stronger statistical support for findings in the Greensboro apiary may have been a result of the higher mite pressure in that location, but it also highlights the potential benefit of measuring hygienic behavior at the colony level rather than making assumptions of resistance based on stock or queen origin alone.

### 4.3 Viral type, prevalence, and co-occurrence patterns

In addition to DWV, our study characterized within-colony dynamics of other viruses, including BRV-1, LSVs, BQCV, SBV, and VDV2. While BRV-1 and VDV2 were prevalent in *Varroa* mites, their titers remained relatively stable and were not significantly impacted by host genotype. VDV2, a member of the *Iflaviridae* family, was first identified as a virus unique to *Varroa* mites and not initially associated with honey bees (Levin et al., 2016). It has since been found to be highly prevalent in *Varroa* mite populations (Chen et al., 2021; Herrero et al., 2019), though it can also be detected in worker honey bees, particularly in cases of heavy *Varroa* infestation (Chen et al., 2021; Lester et al., 2022). The significant seasonal declines in BQCV and SBV, especially in worker bees, may reflect natural viral suppression due to environmental or colony-level factors, as these viruses are often more sensitive to external stressors (Doublet et al., 2024; McAfee et al., 2024; Mondet et al., 2014). LSVs exhibited a unique trend, remaining prevalent across all groups over time but only occasionally differing quantitatively between genotypes. Significant reductions in LSVs were observed in high UBeeO colonies in August and in September mite samples, suggesting that LSVs may be opportunistic viruses more prevalent under weakened immune conditions or elevated viral co-infections (Faurot-Daniels et al., 2020).

Viral co-occurrence network analyses revealed distinct infection patterns in colonies of different genetic backgrounds. In high UBeeO colonies, complex co-infection networks, particularly involving DWV-A/DWV-B and BRV-1/VDV2, suggest a more structured viral ecology. In contrast, viral co-occurrence in low UBeeO and Commercial colonies appeared more stochastic and widespread, potentially reflecting weakened antiviral defense or heightened viral replication due to elevated *Varroa* pressure. Moreover, positive correlations between BRV-1 and VDV2 in *Varroa* mites, particularly *Varroa* mites in high UBeeO colonies, raise questions about the ecological or synergistic relationships among these less-studied viruses. The frequent co-occurrence of LSVs with other viruses in mite samples also suggests the potential role of *Varroa* mites as a mechanical vector, as suggested previously (Shojaei et al., 2023).In conclusion, our results align with previous studies indicating that low *Varroa* mite infestation directly decreases the prevalence and titer of multiple mite-associated honey bee viruses and may also contribute indirectly to lower prevalence of non-mite-associated viruses. Furthermore, our results indicate that *Varroa*-resistant hygienic traits not only reduce mite infestations but are also associated with lower virus titers in honey bees, lower virus titers in mites, and can even impact the co-occurrence and correlation of multiple critically important honey bee viruses. This work underscores the effectiveness and importance of hygienic behavior for achieving honey bee colony pest and disease resistance and highlights the value of selective breeding as a long-term strategy to improve honey bee health and facilitate colony survival.

## Funding information

This research was financed by the honey bee research health grant of the North American Pollinator Protection Campaign (NAPPC) and the United States Department of Agriculture Research Service through Cooperative Agreements #58–6066–9–045 and #58–6066–4–020.

## Ethical approval

This study did not require ethical approval since no human or animal material was involved in the study.

## CRediT authorship contribution statement

**Esmaeil Amiri:** Writing – review & editing, Writing – original draft, Validation, Supervision, Resources, Project administration, Methodology, Investigation, Funding acquisition, Data curation, Conceptualization. **Somayeh Mehrparvar:** Writing – review & editing, Visualization, Software, Methodology, Formal analysis, Data curation. **Bita Valizadeh:** Writing – review & editing, Writing – original draft, Validation, Methodology. **Kaira Wagoner:** Writing – review & editing, Validation, Methodology, Investigation, Data curation, Conceptualization.

## Declaration of competing interest

Kaira Wagoner is co-inventor of the UBeeO assay and is owner of Optera LLC, which developed and now sells UBeeO. To avoid any potential conflict of interest, Dr. Wagoner did not analyze data presented in this study and scored UBeeO assays for experimental colonies prior to viral analysis by co-author Esmaeil Amiri (and thus blind to virus results). The other authors declare that they have no competing financial or non-financial interests that could have appeared to influence the work reported in this paper.

## Data Availability

Data will be made available on request.

## Appendix Supplementary materials

Image, application 1
