# Supplementary material for: Honey bee hygienic selection impacts virus dynamics of both bees and Varroa mites
Source: Virus Res. 2026 Mar 18;367:199715. doi: 10.1016/j.virusres.2026.199715 (PMC13087584; doi:10.1016/j.virusres.2026.199715)
Supplement: Supplementary file 1 [file mmc1.docx]

**Honey bee hygienic selection impacts virus dynamics of both bees and *Varroa* mites**

Esmaeil Amiri^a*^, Somayeh Mehrparvar^a^, Bita Valizadeh^a^, Kaira Wagoner^b^

^a^ Department of Agricultural Sciences and Plant Protection, College of Agriculture and Life Sciences, Delta Research and Extension Center (DREC), Mississippi State University, Stoneville, Mississippi, USA

^b^ Department of Biology, University of North Carolina at Greensboro, Greensboro, NC, USA

* Corresponding Author: Esmaeil Amiri

[ea795@msstate.edu](mailto:ea795@msstate.edu)

# **Supplemental Information**

**Table S1: Primer sequences to detect and quantify viruses both in worker bees and *Varroa* mites.**

| Target | Primer name | Primer sequence (5’-3’) | Product length (bp) | Reference |
| --- | --- | --- | --- | --- |
| DWV-A | DWVnew-F1 | TACTAGTGCTGGTTTTCCTTT | 155 | (Kevill et al., 2017) |
|  | DWVA-R1 | CTCATTAACTGTGTCGTTGAT |  |  |
| DWV-B | DWVnew-F1 | TACTAGTGCTGGTTTTCCTTT | 155 | (Kevill et al., 2017) |
|  | DWVB-R1 | CTCATTAACTGAGTTGTTGTC |  |  |
| LSVs | LSV1-4-F-2157 | CGTGCGGACCTCATTTCTTCATGT | 82 | (Daughenbaugh et al., 2015) |
|  | LSV1-4-R-2239 | CCTCCCACCGCGTGTGC |  |  |
| BQCV | BQCV_F | CGAAGCGTTTTCCGTGG | 182 | (Milone and Tarpy, 2021) |
|  | BQCV_R | GCTGTCGAGAGTCAGAGTT |  |  |
| SBV | SBV_F2_5120 | AATGTCACCCACGAGTGTTG | 125 | (Daughenbaugh et al., 2015) |
|  | SBV_R2_5243 | GCGATGCAACCATACAACTG |  |  |
| VDV2 | 5544F | GGAATTGCGTCGAATTGTCAC | 336 | (Levin et al., 2016) |
|  | 5879R | TTCCTCTCCAGCTTTAGGTGCT |  |  |
| BRV-1 | BRV-qRT-F 1 | ACCCTTTTCGCCTTACCTCG | 183 | (Levin et al., 2017) |
|  | BRV-qRT-R1 | GTCACTGATTGAGCTAGCCGT |  |  |
| RPS5 | RPS5_F | AATTATTTGGTCGCTGGAATTG | 115 | (Evans, 2006) |
|  | RPS5_R | TAACGTCCAGCAGAATGTGGTA |  |  |
| β-actin | FV-β-Actin | GTTCATCGGAATGGAGTCATGCGGT | 108 | (Francis et al., 2013) |
|  | RV β-Actin | CCAGAGAGAACGGTGTTAGCGTACA |  |  |

**Table S2: The prevalence of viruses in worker bees and mite samples in high and low UBeeO colonies at Greensboro apiary. Data are presented as percentage.**

| Viruses | Honey bee samples | | *Varroa* mite Samples | |
| --- | --- | --- | --- | --- |
|  | High UBeeO | Low UBeeO | High UBeeO | Low UBeeO |
| DWV-A | 90.9 | 97.8 | 94.8 | 94.7 |
| DWV-B | 90.9 | 97.8 | 90.8 | 91.4 |
| BQCV | 97.7 | 100 | 11.8 | 13.1 |
| LSVs | 90.9 | 95.7 | 51.1 | 47.2 |
| SBV | 61.4 | 39.1 | 34.9 | 17.8 |
| BRV-1 | 56.8 | 63.0 | 99.1 | 99.1 |
| VDV2 | 15.9 | 10.9 | 99.6 | 99.1 |

**Table S3: The prevalence of viruses in worker bees and *Varroa* mite samples in Pol-line and commercial colonies. Data are presented as percentage.**

| Viruses | Honey bee samples | | *Varroa* mite Samples | |
| --- | --- | --- | --- | --- |
|  | Pol-line | Commercial | Pol-line | Commercial |
| DWV-A | 63.6 | 40.7 | 27.3 | 15.4 |
| DWV-B | 69.1 | 55.9 | 22.7 | 31.6 |
| BQCV | 74.5 | 74.6 | 13.6 | 3.7 |
| LSVs | 98.2 | 100 | 72.7 | 78.7 |
| SBV | 38.2 | 45.8 | 13.6 | 7.4 |
| BRV-1 | 5.5 | 10.2 | 100 | 99.3 |
| VDV2 | - | - | 100 | 99.3 |

**References**

Daughenbaugh, K.F., Martin, M., Brutscher, L.M., Cavigli, I., Garcia, E., Lavin, M., Flenniken, M.L., 2015. Honey bee infecting Lake Sinai Viruses. Viruses 7(6), 3285-3309. <https://doi.org/10.3390/v7062772>

Evans, J.D., 2006. Beepath: an ordered quantitative-PCR array for exploring honey bee immunity and disease. J. Invertebr. Pathol. 93(2), 135-139. <https://doi.org/10.1016/j.jip.2006.04.004>

Francis, R.M., Nielsen, S.L., Kryger, P., 2013. Varroa-virus interaction in collapsing honey bee colonies. PLoS One 8(3), e57540. <https://doi.org/10.1371/journal.pone.0057540>

Kevill, J., Highfield, A., Mordecai, G., Martin, S., Schroeder, D., 2017. ABC assay: method development and application to quantify the role of three DWV master variants in overwinter colony losses of European honey bees. Viruses 9(11), 314. <https://doi.org/10.3390/v9110314>

Levin, S., Galbraith, D., Sela, N., Erez, T., Grozinger, C.M., Chejanovsky, N., 2017. Presence of *Apis Rhabdovirus-1* in populations of pollinators and their parasites from two continents. Frontiers in Microbiology Volume 8 - 2017. <https://doi.org/10.3389/fmicb.2017.02482>

Levin, S., Sela, N., Chejanovsky, N., 2016. Two novel viruses associated with the *Apis mellifera* pathogenic mite *Varroa destructor*. Scientific Reports 6(1), 37710. <https://doi.org/10.1038/srep37710>

Milone, J.P., Tarpy, D.R., 2021. Effects of developmental exposure to pesticides in wax and pollen on honey bee (*Apis mellifera*) queen reproductive phenotypes. Scientific Reports 11(1), 1020. <https://doi.org/10.1038/s41598-020-80446-3>
